# Nervous Women and Gynecology

**Published:** 1916-07

**Authors:** F. S. White

**Affiliations:** San Antonio, Texas


### Nervous Women and Gynecology.

BY F. S. WHITE, M. D., SAN ANTONIO, TEXAS.

In discussing this important question, I propose to do so from the standpoint of the neurologist, rather than that of the surgeon.

I leave the strictly surgical aspect of the subject to the many competent and painstaking surgeons who undoubtedly properly appreciate the conditions which operate to complete the ensemble of symptoms which go to make the nervous woman.

There are certain crises in the life of every woman which tend to develop and accentuate any constitutional defect which may exist?

The first important change which takes place is the establishment of puberty. While this, to be sure, is a physiological process, yet on account of environment, etc., this often becomes an abnormal process, and girls at this important period become variously nervous, excitable, depressed, and often they develop certain characteristics and symptoms which, to the experienced neurologist, indicate that both a physical and mental breakdown is impending. This unfortunate and sad result can often be prevented by proper advice, suggestions and medical treatment. Intelligent treatment at this time may save many girls from a lifetime of invalidism and misery. The family physician is the first to be consulted, but, sad to state, often through ignorance or lack of attention, the case is lightly considered and dismissed with the statement that "all will be well when the menstrual function is established." If this same family physician properly appreciated the gravity of the symptoms, and took into consideration the dire results that may follow, he could divert the case into the proper channels, when good health and a happy life would follow.

These growing girls, knowing that something is wrong and failing to get relief from the family physician, often drift into the hands of charletans and quacks, who may, for personal gain, make mountains out of mole-hills. These unfortunate girls are not hysterical and malingering, but are real and genuine sufferers and are crying for relief, which physicians can give if they have the correct conception of the gravity of the case. Failing to give this relief, the family physician neglects to perform one of the most important duties which he owes to his patrons. Such neglect accounts largely for the majority of cases who seek relief from Christian Scientists, chiropractors and all the fraudulent cults who promise relief to such cases. These case should not be neglected and, if neglected, they have a just grievance against their family physician.

Based upon years of experience in the treatment of such girls, I desire to insist with all the emphasis possible that these cases be given the care, advice and attention which the gravity of the symptoms indicate that they require. So much for the period when the individual is becoming a woman and is entering into the child-bearing period and experiences,—new thoughts, new aspirations, ambitions, and sensations.

The next and last important crisis in woman's life is the time when the child-bearing period is closing and the menopause impending. This is a stage of involution and is fraught with more dangers to the woman, both mentally and physically, than the stage of puberty. She realizes that her period of life when motherhood is possible is closing. She knows that a change is taking place and that afterwards she will be radically different. She often becomes despondent, melancholy and introspective. She often feels that her life has not been just what she, in the buoyancy of youth, hoped it would be; that her race is run and that hereafter she may become a burden on relatives and friends. Her viewpoint changes; her thoughts and feelings change, and at this change suicide of mental breakdown are often the result. At this period, women need more careful and intelligent care and treatment than at puberty. At puberty, she is just entering into womanly life with all the aspirations that its contemplation inspires. At the menopause, she is emerging from this womanly life with a lowered vitality and lessened power to withstand the vicissitudes of oncoming age. To be sure the menopause is in normal women, and, under normal conditions, a physiological process, but it is often accompanied with abnormal symptoms. These symptoms can, to a very large extent, be controlled and modified by the proper measures. At this important crisis, many women break down and lose their mental equilibrium, when this condition supervenes even they are very curable and the great majority can be restored to mental and physical health and continue in good condition through the balance of their days.

Fashions in medicine and surgery change. Not quite, however, as often as the fashions in women's apparel, but change, nevertheless, and we are all nolens volens influenced by these changes. These changes, I am happy to say, usually are in the direction of more intelligent application of the principles of medicine and surgery. There may be nothing new under the sun, but there certainly is great improvement in every line of thought and action over those of our ancestors. There is an inherited inherent quality in every individual, which to a very large extent determines what type of character may be developed. This inherent quality, to be sure, is subject to a very considerable variation of external influences and environment. The individual peculiarities, whether the result directly or indirectly of inheritance or environment, must be taken into careful consideration, especially so when considering a case with neuropathic or psychopathic symptoms. I do not in any manner desire to detract from the magnificent results that have been achieved by modern surgery. In all improvements and advancements, there is always the tendency and danger of going too far one way or the other. There is no doubt but what surgery has attempted too much in a certain type of woman. Recently, however, there are indications of a more rational basis for action being found. I believe that I am safe in saying that women have been unnecessarily deprived of some or all of their generative organs by over-enthusiastic surgeons, but the time and occasion for such errors happily have been relegated to the past. Many women who have inherited an unstable nervous organization will complain of many vague and indefinite symptoms, which often direct the attention of the surgeon to the organs of reproduction. An examination will reveal some pain and soreness on pressure, and this, together with the direction of the patient's attention to these organs, will often magnify the subjective symptoms. It is extremely rare that a neurosis or a psychosis is due primarily to a disturbance of these organs. Abnormal action of these organs, however, just as abnormal action of any other organ or set of organs, may be a factor in the causation of the nervous or mental condition, but what I want to particularly emphasize is that such a condition is usually engrafted upon an unstable nervous organization. The importance of the sexual system has been greatly exaggerated as a cause of the neurosis and psychoses. Scare-head quack advertisements have been so broadly circulated among the people that practically all young people have adopted the idea that any little sexual irregularities, or excesses in youth, lead to irreparable damage to the individual. I would not decry operation on the sufferers from nervous or mental diseases, but what I would discourage is the hope that is often held out that operation will effect a cure; any means or measures which aid in putting the individual into a more normal condition will aid in recovery.

Another idea, which is now of general adoption, and to which I most heartily subscribe, is that no woman shall be robbed of her ovaries when it is possible to save them; if only a part is left, that is certainly better than none. These nervous women, who make the round of physicians' offices, need something more than cold steel. They should be objects of pity rather than of ridicule. Some of them, on account of neuropathic and psychopathic conditions, become obscessed with the idea of being operated upon and will take the great pleasure in relating in the most minute detail the operations which they have undergone. Competent surgeons are more and more appreciating the fact that nervous women cannot be cured of their nervousness by having their ovaries removed. I have seen many unfortunate women who had had all the reproductive organs removed with often an appendectomy and gall bladder operation on the side, but were still possessed of an abnormal and unstable nervous organization, which was bequeathed to them by their ancestors.

Another point which should be given the most careful consideration is the after-effects on the woman when the reaction comes, and she awakens to the realization that she has been unsexed, and that she is no longer capable of becoming a mother. Some of the most miserable creatures that it has been my misfortune to see are just such cases. The surgical conscience should be active and the most careful consideration should be given to the individual woman to be operated on, and the probable ultimate results of such an operation, not only from the physical standpoint, but as to the effect upon the mentality.

Now, I have not thought it best to go into details in describing the different psychoses and neuroses met with, but have endeavored to briefly outline the underlying causes which often lead cases to the surgeon, and to call attention to the fact that surgery cannot correct the inherent defects of an unstable nervous organization.

["Dementia Precox" will be the subject of Dr. White's next paper. Don't fail to read it. Remember, Dr. White is "an insanity expert," and out of the fullness of years of experience and practice he is preparing these articles especially for the readers of the Texas Medical Journal.—Editor.]

